# Acquired factor XIII deficiency in two patients with bleeding events during veno-venous extracorporeal membrane oxygenation treatment

**DOI:** 10.1007/s10047-019-01148-w

**Published:** 2019-12-13

**Authors:** Asami Ito, Yoshiaki Iwashita, Ryo Esumi, Ken Sasaki, Masahiro Yukimitsu, Takafumi Kato, Eiji Kawamoto, Kei Suzuki, Hiroshi Imai

**Affiliations:** 1 Mie University Hospital, Tsu, Japan; 2 Shimane University Hospital, Izumo, Japan

**Keywords:** Factor XIII, VV-ECMO, Factor XIII deficiency

## Abstract

We report two cases of acquired factor XIII deficiency with bleeding events during veno-venous extracorporeal membrane oxygenation (ECMO). Case 1: A 76-year-old man diagnosed with aspiration pneumonia after near-drowning was started on ECMO. Later, the patient presented with hemoptysis and anemia. Blood tests showed a decreased factor XIII activity of 29%. Although the patient recovered after receiving 1200 International Units of factor XIII concentrate, the patient had another episode of decreased factor XIII activity and bloody stool and was treated again with factor XIII concentrate. Case 2: A 48-year-old female diagnosed with pneumonia was started on ECMO. Soon after, she presented with hemoptysis and anemia. Blood tests showed a decreased factor XIII activity of 39%. The patient was treated with 720 IU of factor XIII concentrate with good recovery. Acquired factor XIII deficiency cannot be detected by routine coagulation tests, therefore it may be under-diagnosed in the ICU. Detection of acquired factor XIII deficiency is essential when treating a bleeding ECMO patient.

## Introduction

Veno-venous (VV) extracorporeal membrane oxygenation (ECMO) can be a successful treatment for certain patients. While use of ECMO is becoming more widely used among institutions, bleeding events are not uncommon in the ICU. Furthermore, bleeding events lead to a low survival rate due to hemodynamic instability, massive transfusion and prolonged ECMO treatment [1, 2]. We herein report two VV-ECMO patients with uncontrollable bleeding events and acquired factor XIII deficiency, who were successfully treated with factor XIII concentrate.

## Case 1

**Fig. 1:**
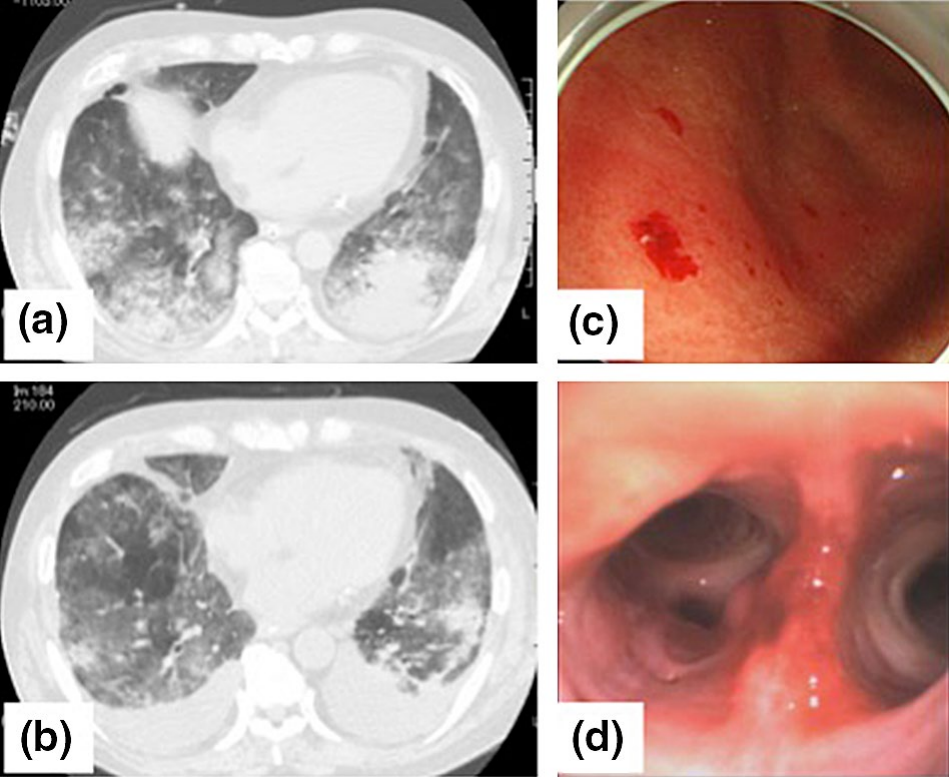
Case 1: **a** CT scan on arrival and **b** on the day of the second run. **c** Gastrointestinal endoscopy shows micro-hemorrhage. **d** Bronchoscopy shows bloody sputum, blood clots and bleeding from the bronchial wall

**Fig. 2:**
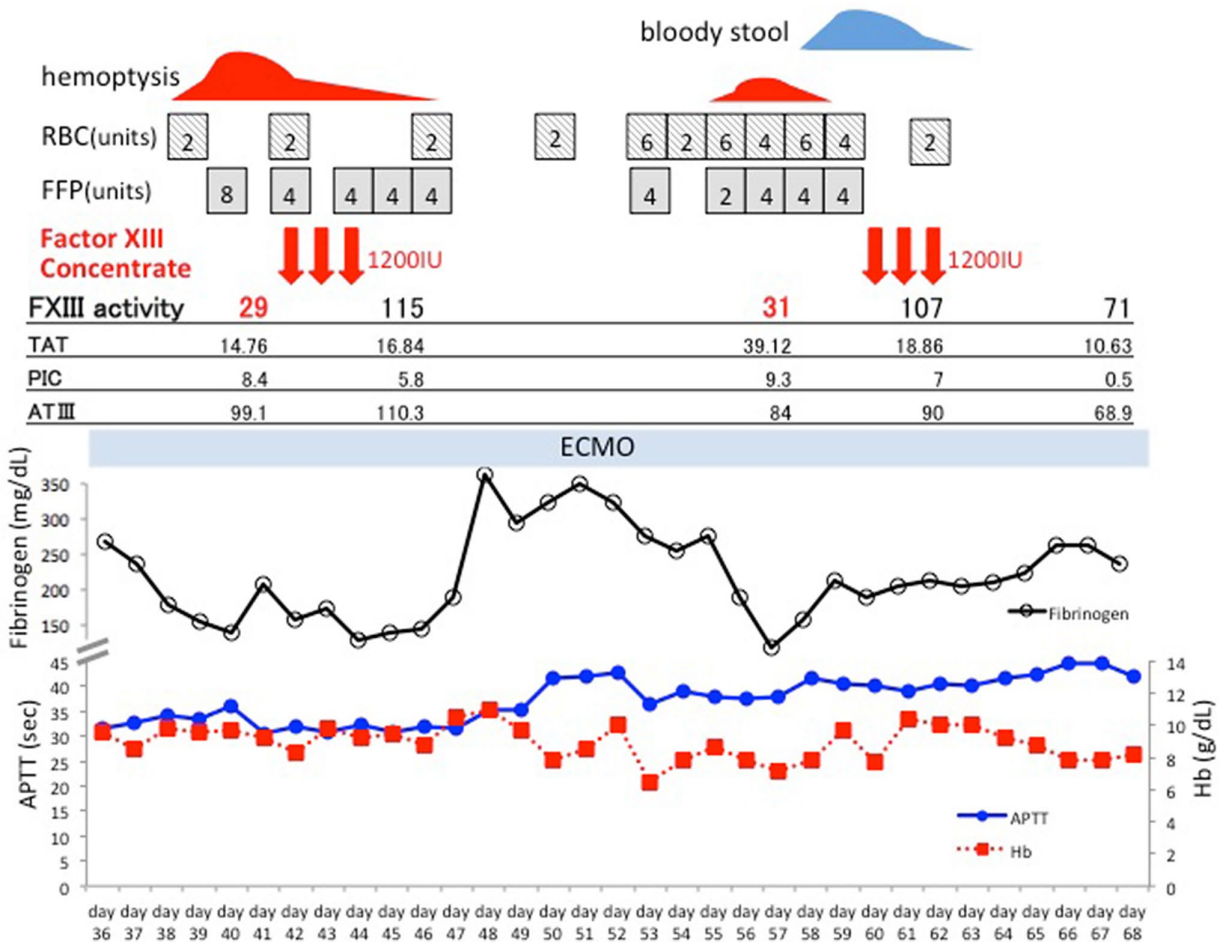
Clinical course of the patient (Case 1)

A 76-year-old man was transported to the emergency room for respiratory failure due to near drowning. CT scan showed widely distributed bilateral infiltration (Fig. 1a). The patient had severe respiratory failure despite mechanical ventilation settings of PEEP 12 cmH_2_O and FiO_2_ 1.0, with a PaO_2_/FiO_2_ (*P/F*) ratio of 82. VV-ECMO was initiated shortly after arrival. An access cannula was placed in the right femoral vein, and a return cannula was placed in the right internal jugular vein. Initial ECMO circuit flow was set at 2000–2500 rpm, 3.5–4.5 L/min. ECMO circuit FiO_2_ was set at 1.0 during the entirety of support. The patient improved and was weaned off of ECMO on the 5th day. However, his respiratory failure relapsed due to volume overload and infection (Fig. 1b) leading to a second run of ECMO on the 15th day. On the 40th day, we observed bleeding from the cannulation site and hemoptysis, along with anemia. Bronchoscopy revealed hemorrhage from the bronchial wall and blood clots (Fig. 1d). Heparin infusion was discontinued. APTT and PT-INR ranged from 30.6–42.7 s and 0.97–1.09, respectively. Additional coagulation tests showed a factor XIII activity of 29%. The patient received 1200 International Units (IU) of factor XIII concentrate, by which clinical symptoms and blood tests promptly recovered, with a factor XIII activity of 115% after treatment. However, a few days later, the patient had another bleeding episode of bloody stool. This time factor XIII activity was 31%, which recovered to 107% after the same dose of factor XIII concentrate. Gastrointestinal endoscopy revealed micro-hemorrhage due to chronic gastritis (Fig. 1c). The patient’s clinical course and coagulation data are shown in Fig. 2. Despite recovery from continuous bleeding, he died on the 88th day due to irreversible acute respiratory distress syndrome (ARDS).

## Case 2

**Fig. 3:**
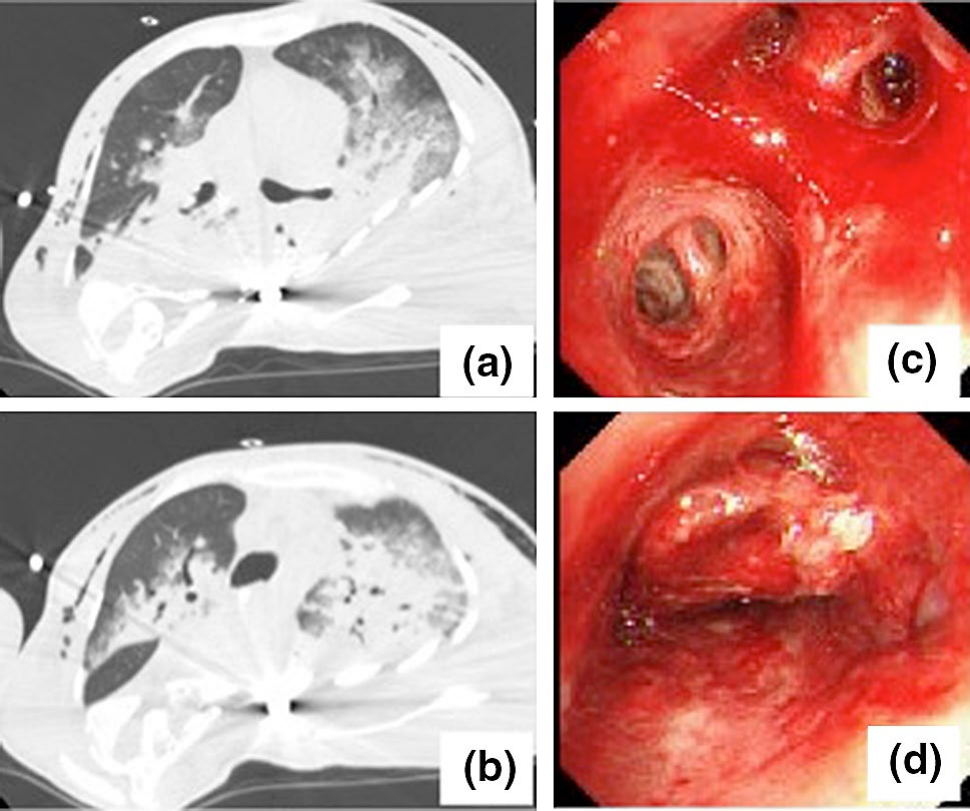
Case 2: **a**, **b** CT scan on arrival. **c**, **d** Bronchoscopy after hemoptysis shows bleeding and blood clot occluding the left main bronchus

**Fig. 4:**
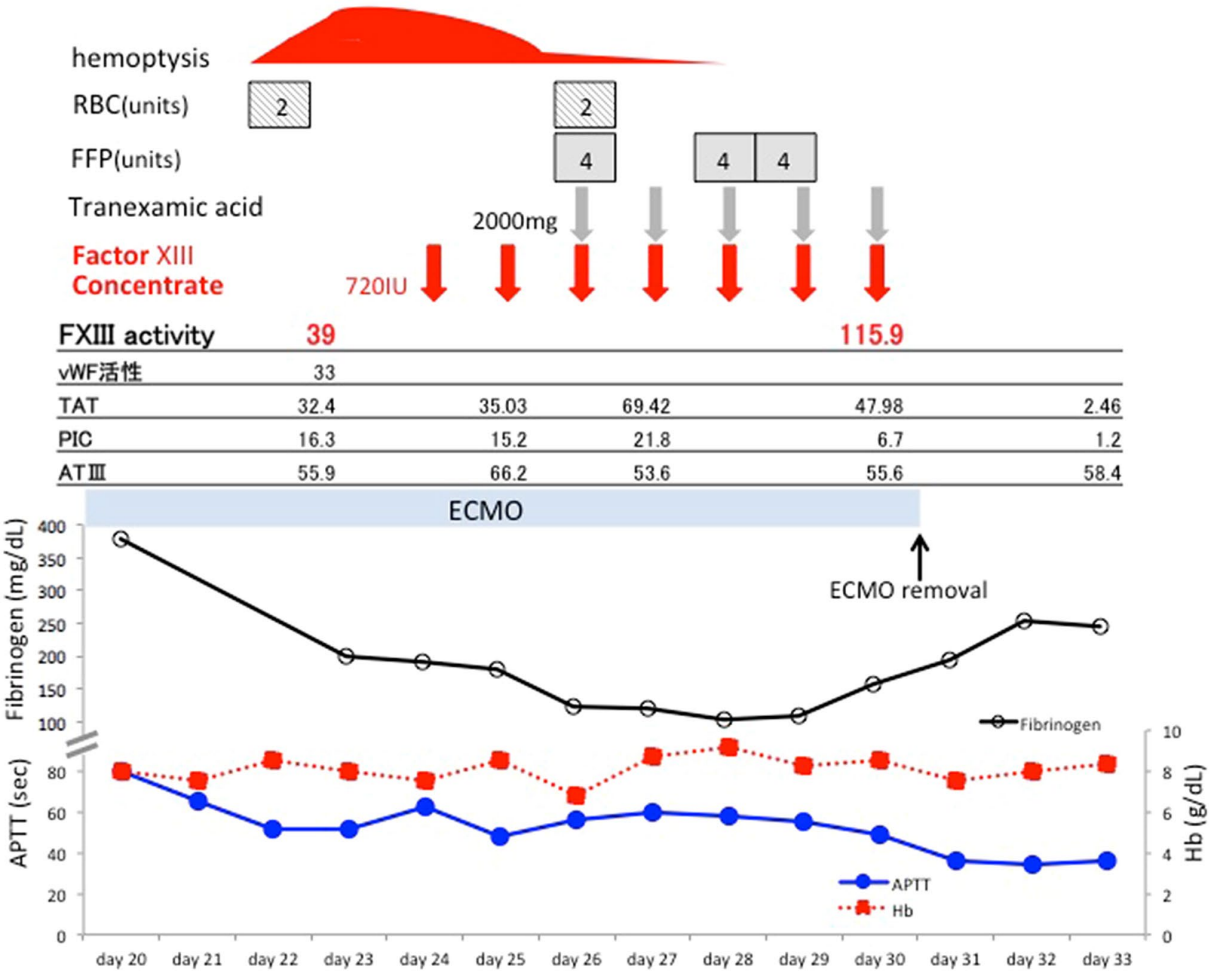
Clinical course of the patient (Case 2)

A 48-year-old female was transported to the emergency room with a complaint of dyspnea. The patient had a history of surgery for scoliosis. On arrival, the patient presented with hypotension, low consciousness level with a GCS of E3V4M5, and severe hypoxemia. CT scan showed bilateral infiltration and subcutaneous emphysema (Fig. 3a, b). The diagnosis of pneumonia complicated with ARDS and multiple organ failure was made. P/F ratio was 75 despite prone positioning and airway pressure release ventilation (APRV). VV-ECMO was initiated 6 h after admission. Cannula placement and initial ECMO setting were similar to those described for Case 1. Weaning of ECMO was hindered due to alveolar hemorrhage and atelectasis from blood clot formation, as confirmed on bronchoscopy (Fig. 3c, d). Blood test showed APTT 51.3 s, PT-INR 1.21, fibrinogen 193 mg/dL, TAT 32.3 ng/mL, PIC 16.3 μg/mL, factor XIII activity 39%, von Willebrand factor activity 33%, von Willebrand factor antigen 102%. Heparin infusion was discontinued. APTT and PT-INR ranged from 47.7–60.3 s and 1.13–1.21, respectively. The patient received 720 IU of factor XIII concentrate for 7 days, whereupon factor XIII activity improved to 115% along with improvement of clinical symptoms. Since the patient’s data indicated a hyperactivated state of fibrinolysis, we infused 2000 mg of tranexamic acid for five consecutive days. However, we were careful of any signs of hypercoagulability, such as elevated d-dimers and oxygenator thrombosis, which fortunately did not occur. The patient’s clinical course and coagulation data are shown in Fig. 4. The patient was weaned off of ECMO on the 30th day.

## Discussion

We report two cases of acquired factor XIII deficiency with bleeding events during ECMO, treated successfully with factor XIII concentrate. This case highlights the following two important clinical insights: (1) Acquired factor XIII deficiency cannot be detected by routine coagulation tests, thereby uncontrollable bleeding should be considered highly suspicious despite normal routine coagulation tests. (2) There is limited data on acquired factor XIII deficiency in ECMO patients and the optimal monitoring and treatment goals remain unclear.

Severe bleeding events that occur during VV-ECMO treatment are critical and may potentially impact the odds of survival. Such bleeding events during ECMO are frequent in the ICU; serious bleeding events are reported in 50–60% of patients [1, 2]. In a majority of cases, systemic anti-coagulation therapy is administered in order to prevent thromboembolic complications, which would increase the risk of bleeding.

Mechanical shear stress derived from the circuit and blood flow may also play a part in coagulation disorders during extracorporeal circulation. It is estimated that ECMO extracorporeal blood undergoes high shear stress, leading to the uncoiling of von Willebrand factor resulting in a state of thrombosis, fibrinolysis, and impaired platelet function [3]. Several studies have reported acquired von Willebrand syndrome in patients undergoing cardiopulmonary bypass and VA/VV ECMO [4, 5].

Compared to acquired von Willebrand syndrome in extracorporeal life support (ECLS) patients, studies on acquired factor XIII deficiency are few. Plasma factor XIII circulates in the blood in the form of a complex with fibrinogen. Factor XIII plays a prominent role in clot stabilization by ligation of adjacent fibrin monomers near the end of the coagulation cascade. Acquired factor XIII deficiency in postoperative patients is related to excessive blood loss and the higher demand of blood transfusion [6]. It is estimated that acquired factor XIII deficiency in these cases occurs due to dilution, consumption, and loss. In cardiac surgery patients undergoing cardiopulmonary bypass, studies have shown that extracorporeal circulation leads to a decrease in factor XIII plasma levels. One study showed that the factor XIII level was already decreased 2 h after surgery [7]. The prevalence of factor XIII deficiency in ECMO patients is unclear, however, in a small sampled study, it was reported that 88% of VV-ECMO patients showed a decrease of factor XIII activity [8]. The direct mechanism of decreased factor XIII activity remain to be elucidated. In the two cases presented here, both patients were in a state of systemic inflammation, and also required high volume fluid and transfusion, which would put them at a high risk for acquired factor XIII deficiency due to consumption and loss. It should be noted that although plasma concentration of factor XIII required for hemostasis is low (0.1–0.5 U/L), the recovery rate of factor XIII after transfusion of fresh frozen plasma is highly unstable (5–100%) [9]. We estimate that mechanical injury due to ECMO combined with the preexisting patient factors may have led to the acquired factor XIII deficiency. In addition, the subcutaneous emphysema in the second case may have been related to a low factor XIII activity, as factor XIII is essential for wound healing. Since acquired factor XIII deficiency cannot be detected by routine coagulation tests, it may be an under-diagnosed condition that may critically impact survival.

As there are few reports or studies of acquired factor XIII deficiency in ECMO patients, optimal management and treatment remain unknown. Further studies are warranted to elucidate the mechanisms of acquired factor XIII deficiency during ECMO, and to establish an effective algorithm to manage and treat bleeding ECMO patients.
